# Secretory leukocyte protease inhibitor regulates human periodontal ligament cell production of pro-inflammatory cytokines

**DOI:** 10.1007/s00011-017-1062-2

**Published:** 2017-06-08

**Authors:** Daniel Svensson, Alexandra Aidoukovitch, Emma Anders, Daniel Jönsson, Daniel Nebel, Bengt-Olof Nilsson

**Affiliations:** 1 Department of Experimental Medical Science, Lund University, BMC D12, 221 84 Lund, Sweden; 2 Folktandvården Skåne, Malmö, Sweden

**Keywords:** Cytokines, NF-κB, Periodontal ligament cells, Periodontitis, Secretory leukocyte protease inhibitor

## Abstract

**Objective:**

Regulation of immune-like cell properties of periodontal ligament (PDL) cells is not understood. We investigate the importance of secretory leukocyte protease inhibitor (SLPI) for production of pro-inflammatory cytokines in human PDL cells.

**Materials and methods:**

PDL cells were isolated from teeth extracted for orthodontic reasons. Cellular location of SLPI was investigated by immunocytochemistry. Cytokine transcript and protein expression were assessed by quantitative real-time RT-PCR and Western blotting. SLPI gene activity was knocked-down by siRNA. NF-κB signaling was assessed by measuring IκBα, and phosphorylated p65 and p105 protein expression.

**Results:**

PDL cells showed cytoplasmic expression of SLPI. Cellular expression level of SLPI negatively correlated to LPS-induced stimulation of IL-6 and MCP-1. Both SLPI gene activity and protein were reduced by about 70% in PDL cells treated with SLPI siRNA compared to cells treated with non-coding construct. Treatment with SLPI siRNA was associated with up-regulation of both basal and LPS-stimulated IL-6, MCP-1 and TLRs mRNA expression. The up-regulation of MCP-1 transcript in SLPI siRNA-treated cells was confirmed on protein level. SLPI siRNA-treatment enhanced the phosphorylated NF-κB p105 protein expression.

**Conclusions:**

SLPI regulates PDL cell pro-inflammatory cytokine expression and modulates NF-κB signaling, suggesting that SLPI governs the immune cell-like properties of PDL cells.

## Introduction

Periodontal ligament (PDL) cells are fibroblast-like cells, but they also show osteoblastic features such as expressing bone marker proteins and alkaline phosphatase activity and mineralized nodule formation [1, 2]. The PDL cells produce pro-inflammatory cytokines upon stimulation with bacterial endotoxins such as lipopolysaccharides (LPS), suggesting that they may act as immune cells and contribute to initiation and progress of periodontal inflammation via this mechanism [3]. Interestingly, the nutrient and hormone vitamin D have recently been shown to attenuate PDL cell production of pro-inflammatory cytokines, suggesting that it may influence the development of periodontal inflammation through this mechanism [4, 5]. Although pro-inflammatory cytokines such as the interleukin-6 (IL-6) and IL-1β are regarded as osteolytic factors in periodontitis, there are reports suggesting that IL-6 and IL-1β also may drive osteogenic differentiation of PDL cells [6, 7]. Thus, PDL cell production of cytokines may both promote the inflammatory reaction and also enhance healing and tissue regeneration. Importantly, since the regulatory mechanisms which determine the functional properties of PDL cells are poorly understood, more information is needed to clarify this issue.

IL-6 is thought to be involved in the immune response in many inflammatory diseases including periodontitis [8, 9]. Another prominent cytokine associated with periodontitis is the chemokine monocyte chemoattractant protein-1 (MCP-1) attracting monocytes to the site of inflammation [10, 11]. In human PDL cells, LPS-stimulation strongly enhances the production of both IL-6 and MCP-1 [12]. LPS enhances cytokine expression primarily via binding to the Toll-like receptors (TLR4 and TLR2), and this complex then activates signaling via the nuclear factor kappa light chain enhancer of activated B cells (NF-κB) pathway leading to transcription of cytokine genes [13, 14].

Secretory leukocyte protease inhibitor (SLPI) is a potent inhibitor of serine proteases [15]. SLPI is found in saliva, nasal secretion and expressed by oral and bronchial epithelial cells and also by human HaCaT keratinocytes [16–20]. Intracellular SLPI translocates from cytoplasm to nucleus and binds to NF-κB binding sites in the promoter region of the TNF-α and IL-8 (CXCL-8) genes [21]. Thus, intracellular SLPI may antagonize NF-κB activation of cytokine genes through this mechanism. Furthermore, SLPI seems to prevent the degradation of nuclear factor of kappa light polypeptide gene enhancer in B cells inhibitor alpha (IκBα), another pathway for SLPI-induced inhibition of NF-κB activation [21–23]. The secretory form of SLPI has been shown to interact with LPS and thus inhibit the binding of LPS to its cell surface receptors [24]. Interestingly, Menckeberg et al. [25] recently demonstrated that SLPI determines the microbial responsiveness of human buccal epithelial cells, and, further, that SLPI is a player in the development of the hyporesponsiveness to microbial stimuli that develops at birth.

The objective of the present study was to investigate the hypothesis that SLPI governs pro-inflammatory cytokine expression in human PDL cells and, thus, determines their sensitivity to LPS and, furthermore, to assess underlying mechanisms with focus on the NF-κB signaling pathway. Importantly, we demonstrate in the present study that SLPI negatively regulates both basal and LPS-stimulated IL-6 and MCP-1 expression and that this effect is associated with modulation of TLR2 and TLR4 expression and NF-κB signaling, offering a novel mechanism for the regulation of PDL cell production of pro-inflammatory cytokines.

## Materials and methods

### Cells and cell culture

The PDL cells were obtained from 6 healthy patients on no medication (three boys and three girls, 12–16 years of age) referred for extraction of premolars on orthodontic indications. Both the patients and their parents were informed orally and in writing and a written consent was signed. This procedure was approved by the Human Ethical Committee at Lund University, Lund, Sweden, and the work was carried out in accordance with The Code of Ethics of the World Medical Association (Declaration of Helsinki). The PDL cells were isolated as described previously by Nebel et al. [26]. Briefly, the periodontal ligament was carefully scraped-off only from the middle third of the root surface to avoid contamination from gingival and apical tissues. The tissue explants of periodontal ligament were washed in phosphate-buffered saline (PBS), seeded in culture flasks in Dulbecco’s modified Eagle’s medium (DMEM) supplemented with antibiotics (penicillin 50 U/ml, streptomycin 50 µg/ml) and fetal calf serum (10%), and then the culture flasks were placed in a water-jacketed cell incubator at 37 °C under 5% CO_2_ in air. The PDL cells were allowed to migrate from the explants, and after reaching confluence the cells were trypsinized, counted in a LUNA**™** automatic cell counter (Logos Biosystems, Annandale, VA, USA) and then re-seeded at a density of 100,000 cells/ml. The PDL cells showed a spindle-like shape characteristic to fibroblast-like cells, assessed using a phase-contrast microscope (Olympus CKX41, Olympus Europa GmbH, Hamburg, Germany), and they responded identically to stimulation with LPS irrespective of the donor. Earlier studies conclude that PDL cells obtained from premolars extracted for orthodontic reasons in young and healthy individuals show identical morphology and functional characteristics independent of donor [27, 28]. The human epithelial, keratinocyte HaCaT cell line was from CLS Cell Lines Service GmbH (Eppelheim, Germany). The PDL cells and HaCaT cells were cultured in a mixture of Ham’s F12 and DMEM cell culture medium (1:1) supplemented with antibiotics and 10% fetal calf serum and used for experiments at 80% confluence in passages 2–5 for PDL cells and 2–20 for the HaCaT cells. The cell plates with PDL cells and HaCaT cells were placed in a water-jacketed cell incubator at 37 °C under 5% CO_2_ in air.

### Transfection with siRNA for SLPI

Two SLPI siRNA constructs (Hs_SLPI_5 and Hs_SLPI_7 FlexiTube siRNA) and one non-targeting control construct (NC, Silencer^®^ Negative Control No. 1 siRNA) were purchased from Qiagen (Venlo, The Netherlands) and Thermo Fisher Scientific (Waltham, MA, USA), respectively, and transfected into PDL cells using Oligofectamine™ transfection reagent (Thermo Fisher Scientific). The RNA/Oligofectamine mix was prepared in Opti MEM medium (Thermo Fisher Scientific). The cells were treated with either SLPI siRNA (50 nM of each, final concentration) or non-coding (NC), scrambled construct (100 nM) for 96 h according to the manufacturer’s instructions. The PDL cells, treated with either SLPI siRNA or NC, were stimulated with or without LPS (1 µg/ml LPS, *Escherichia coli* 0111:B4 LPS; Sigma-Aldrich, St Louis, MO, USA) during the last 30 min or 24 h of the 96 h transfection period. The LPS was dissolved in PBS. Controls received PBS as vehicle as appropriate.

### Quantitative real-time RT-PCR

Total RNA from PDL cells was extracted and purified using miRNeasy mini kit (Qiagen). RNA concentration and quality were determined using a NanoDrop 2000C spectrophotometer (Thermo Fisher Scientific). The samples were subjected to one-step quantitative real-time RT-PCR measurements using QuantiFast SYBR Green RT-PCR kit (Qiagen), QuantiTect primer assays (Qiagen) on a Step One Plus real-time thermal cycler (Applied Biosystems, Waltham, MA, USA). Gene expression was calculated using the delta CT method with GAPDH as reference gene according to Pfaffl [29]. Each sample was analyzed in duplicate. Primers for IL-6 (Hs_IL6_1_SG), MCP-1 (Hs_CCL2_1_SG), TLR2 (Hs_TLR2_1_SG), TLR4 (Hs_TLR4_1_SG), SLPI (HS_SLPI_1_SG) and GAPDH (Hs_GAPDH_2_SG) were purchased from Qiagen. These primers from Qiagen are validated and show very high (~100%) efficiency according to the manufacturer’s instructions.

### Immunocytochemistry

The PDL cells were cultured on coverslips and used for immunocytochemistry after reaching 60% confluence. The cells were washed with PBS, fixed in 4% paraformaldehyde and then permeabilized in 0.2% Triton X-100 for 10 min. The non-specific binding sites were blocked with 2% BSA and thereafter the cells were incubated overnight at 4 °C with a polyclonal antibody against SLPI (Novus Biologicals, cat. no. AF1274, Littleton, CO, USA), raised in goat, at a concentration of 5 µg/ml. The following day, cells were incubated for 2 h with secondary Alexa-Fluor 488 Donkey Anti-Goat IgG Antibody (Thermo Fisher Scientific, cat. no. A-11055) at a dilution of 1:500. The coverslips were washed with PBS, rinsed with water, and then mounted using Fluoroshield histological mounting medium with DAPI (Sigma-Aldrich). DAPI was included in the mounting medium to stain the nuclei. The SLPI immunoreactivity and DAPI staining were analyzed using an Olympus BX60 fluorescence microscope with appropriate filter setting. No SLPI immunoreactivity was observed after omission of the primary SLPI antibody.

### Western blotting

The cells were washed with PBS and then lysed in SDS sample buffer composed of Tris/HCl 62.5 mM, pH 6.8, 2% SDS, 10% glycerol and 1 mM phenylmethanesulfonyl fluoride. The samples were sonicated for 10 s, boiled (5 min) and then centrifuged at 16,000 ***g***. Supernatants were collected and the total protein concentration was determined in each sample using a Bio-Rad DC protein assay kit (Bio-Rad, Hercules, CA, USA) to assure equal protein loading for each lane. Then, the samples were supplemented with 2-mercaptoethanol (5%) and bromophenol blue (10‰), both from Sigma-Aldrich, before loaded on the gel. Proteins were separated by SDS/PAGE using Criterion TGX 4–15% or 18% precast gels from Bio-Rad with equal load of protein (60 µg) in each lane. After separation, proteins were transferred to 0.2 µm nitrocellulose membranes using Trans-Blot Turbo transfer system (Bio-Rad). Membranes were blocked with 1% casein and then incubated overnight with antibodies against SLPI (Novus Biologicals, cat. no. AF1274, Littleton, CO, USA), raised in goat, at a concentration of 1 µg/ml, MCP-1 (Abcam, cat. no. ab9669, Cambridge, United Kingdom), raised in rabbit, at a concentration of 0.6 µg/ml, IκBα (Cell Signaling, cat. no. 9242S, Danvers, MA, USA), raised in rabbit, at a dilution of 1:1000, phosphorylated NF-κB p65 (Cell Signaling, phospho S536, cat. no. 3031S), raised in rabbit, at a dilution of 1:1000, phosphorylated NF-κB p105 (Cell Signaling, phospho S933 (18E6), cat. no. 4806S), raised in rabbit, at a dilution of 1:1000 and glyceraldehyde 3-phosphate dehydrogenase (GAPDH, mouse monoclonal, clone 6C5, Merck Millipore, cat. no. MAB374, Merck Millipore, Billerica, MA, USA), raised in mouse, at a dilution of 1:5000. After incubation with primary antibodies, the membranes were carefully washed in Tris-buffered saline with Tween 20 (TBS-T). The immunoreactive bands were visualized by chemiluminescence using horseradish peroxidase (HRP)-conjugated secondary anti-rabbit, anti-goat and anti-mouse antibodies and SuperSignal West Femto chemiluminescence reagent (Thermo Fisher Scientific). The SLPI, MCP-1, IκBα and phosphorylated NF-κB p65 and p105 immunoreactive bands were analyzed by photodensitometric scanning and normalized to GAPDH which served as internal control.

### Statistics

Summarized data are presented as mean ± SEM. Statistical significance was calculated using Student’s *t* test for single comparisons between two groups and ANOVA with Tukey´s Multiple Comparison test for post hoc analysis for multiple comparisons. *P* values <0.05 were considered significant.

## Results

### Human PDL cells express SLPI

**Fig. 1:**
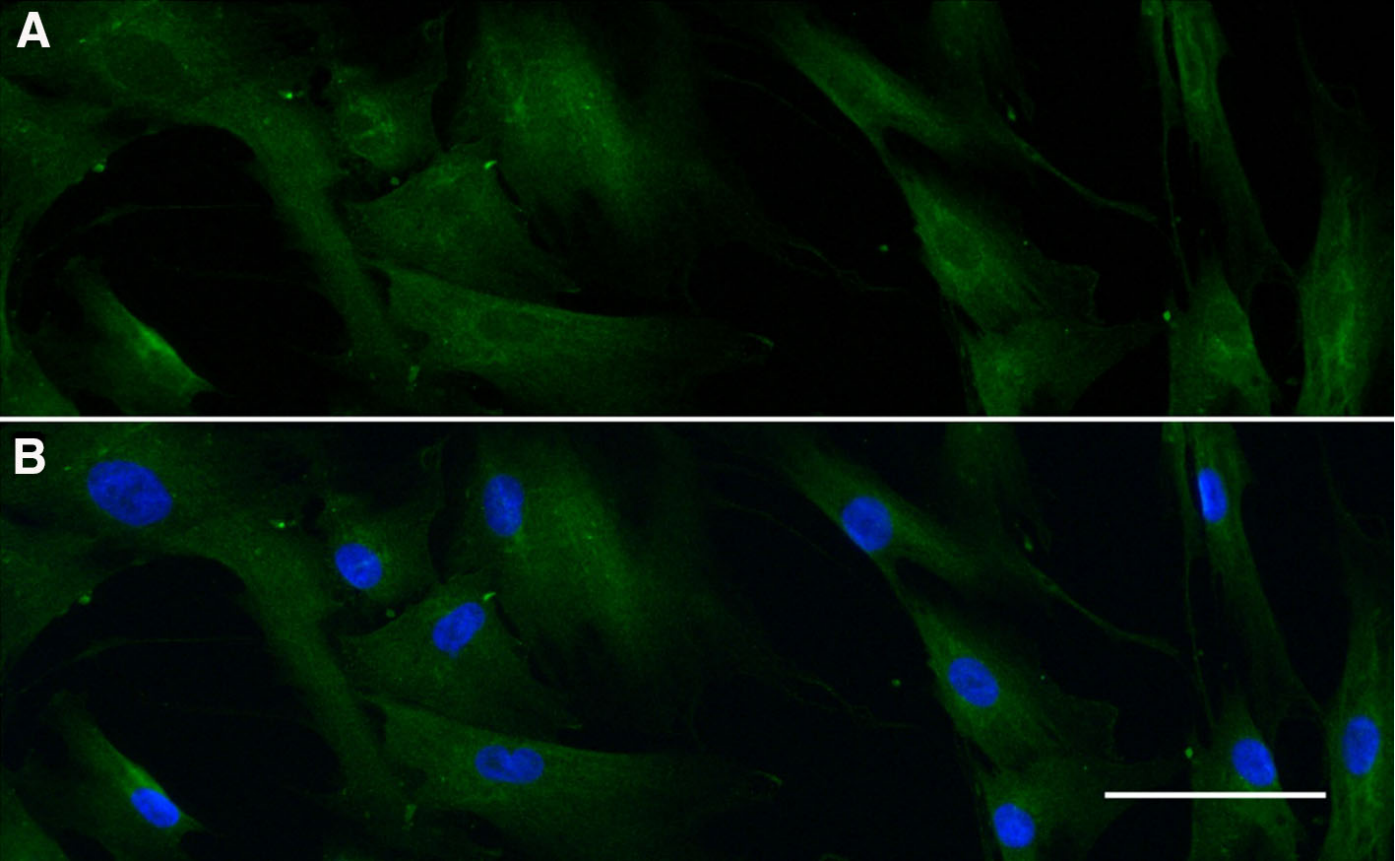
Human PDL cells show cytoplasmic expression of SLPI immunoreactivity (**a**, **b**, *green*). **a**, **b** Show the same cells with appropriate filter settings visualizing either SLPI immunoreactivity alone in *green* (**a**) or both SLPI immunoreactivity and the nuclear marker DAPI in *green* and *blue*, respectively (**b**). *Bar* in *panel* **b** represents 20 µm and applies to *both panel* **a** and **b**

**Fig. 2:**
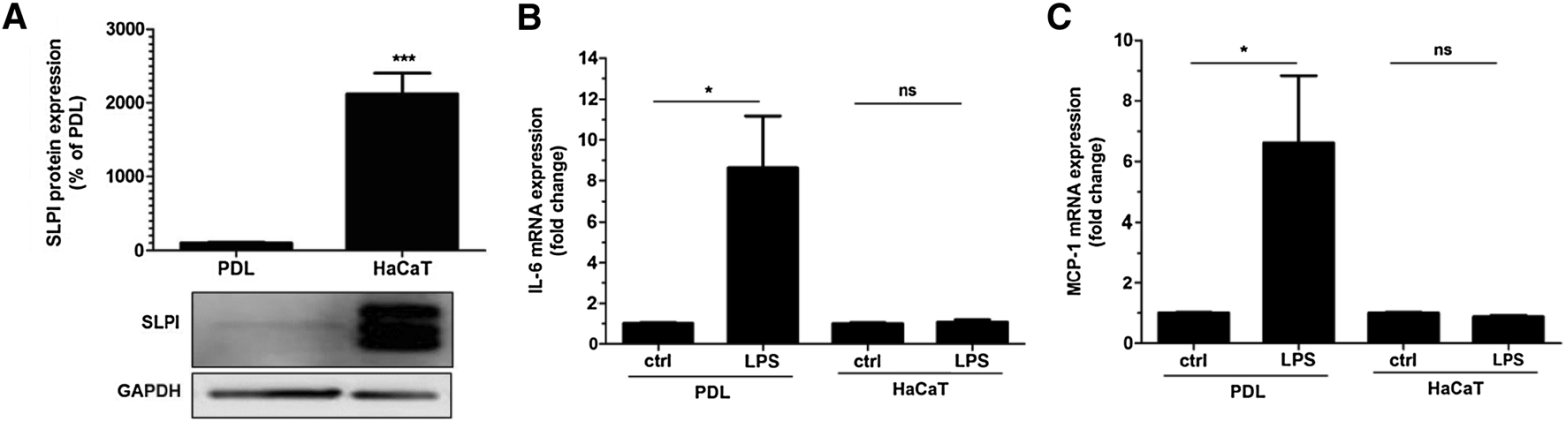
LPS enhances IL-6 and MCP-1 mRNA expression in PDL but not HaCaT cells. The SLPI protein was determined in PDL and HaCaT cells by Western blotting (**a**). The PDL cells and HaCaT cells were cultured under identical culture conditions. The SLPI protein is detected at the expected molecular weight (~14 kDa). The relative concentration of SLPI protein was assessed by densitometry and normalized to GAPDH serving as internal control. PDL and HaCaT cells, cultured under identical conditions, were stimulated with LPS (1 µg/ml) for 24 h and IL-6 and MCP-1 mRNA expression determined by quantitative real-time RT-PCR (**b**, **c**). Values are presented as mean ± SEM of 3-6 observations in each group. * and *** represent *P* < 0.05 and *P* < 0.001, respectively. *ns* non-significant

We investigated the cellular expression of SLPI protein in PDL cells by immunocytochemistry (Fig. 1). SLPI immunoreactivity was observed abundantly in the cytoplasm of all cells (Fig. 1a, b). Some enrichment of SLPI immunoreactivity was observed in the perinuclear region of the cytoplasm. No or very weak nuclear SLPI immunoreactivity was detected (Fig. 1a, b). To assess the expression level of SLPI in PDL cells, we measured SLPI protein expression in both human PDL and HaCaT cells by Western blotting. We compared SLPI protein expression in PDL cells with that of human HaCaT epithelial cells, since HaCaT cells and primary human oral epithelial cells are known to express and secrete SLPI and, thus, HaCaT cells can be regarded as a positive control for SLPI [18, 20, 25]. Western blotting showed that the PDL cells expressed an immunoreactive band at a position corresponding to the correct molecular weight for SLPI (Fig. 2a). In HaCaT cells, included as a positive control, a triplet of immunoreactive bands was observed (Fig. 2a). For summarized data, only the middle band, corresponding to the SLPI immunoreactive band in the PDL cells, was evaluated. Comparison of the SLPI immunoreactive band in PDL and HaCaT cells showed that the expression level of SLPI was about 20 times higher in HaCaT compared to PDL cells (Fig. 2a). The seemingly stronger SLPI immunoreactivity observed in immunocytochemistry compared to Western blotting for PDL cells may suggest that the SLPI antibody is better suited for immunocytochemistry than Western blotting. Next, we assessed if the expression level of SLPI protein in PDL and HaCaT cells, respectively, corresponds to their responsiveness to LPS. We treated PDL and HaCaT cells with LPS (1 µg/ml) for 24 h and determined their expression of the pro-inflammatory cytokines IL-6 and MCP-1. Stimulation with LPS had no effect neither on IL-6 nor MCP-1 transcript level in HaCaT cells but increased both IL-6 and MCP-1 mRNA severalfold in the PDL cells (Fig. 2b, c). Thus, LPS-induced stimulation of IL-6 and MCP-1 expression correlates negatively with cellular expression level of SLPI.

### Knockdown of SLPI by siRNA enhances PDL cell gene expression of IL-6, MCP-1 and TLRs

**Fig. 3:**
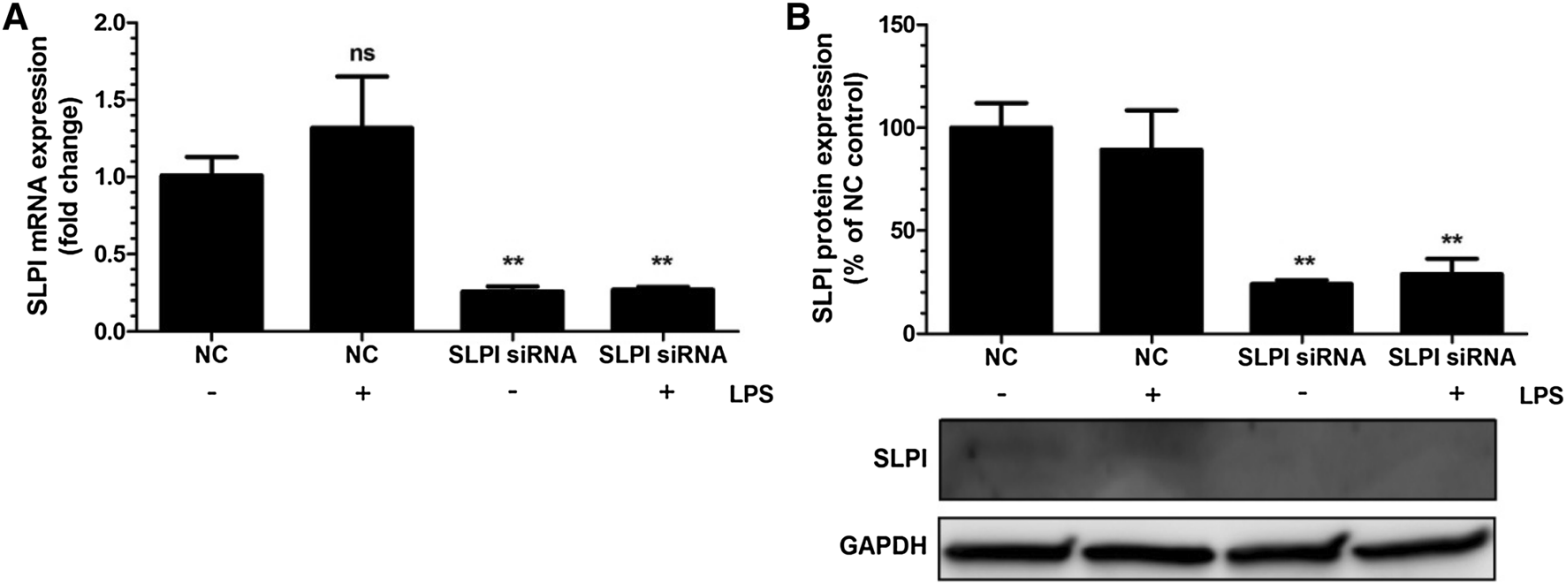
Treatment with siRNA for SLPI reduces both SLPI mRNA (**a**) and SLPI protein (**b**) in PDL cells. The cells were treated with either SLPI siRNA or non-targeting control (NC) for 96 h. LPS (1 µg/ml) was included during the last 24 h of the 96 h transfection period. Total RNA and protein were isolated and mRNA (**a**) and protein (**b**) expression for SLPI determined by quantitative real-time RT-PCR and Western blotting, respectively. Values are presented as mean ± SEM of 3–6 observations in each group. ** represents *P* < 0.01 versus NC in the absence of LPS. *ns* non-significant

**Fig. 4:**
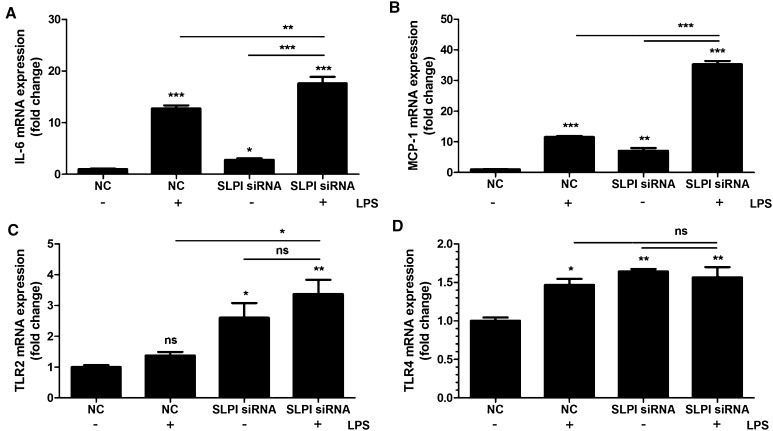
Knockdown of SLPI by treatment with siRNA causes up-regulation of both basal and LPS-induced PDL cell cytokine and TLR expression (**a**–**d**). The PDL cells were treated with either SLPI siRNA or NC for 96 h. LPS (1 µg/ml) was included during the last 24 h of the 96 h transfection period. Total RNA was isolated and mRNA expression for IL-6 (**a**), MCP-1 (**b**), TLR2 (**c**) and TLR4 (**d**) determined by quantitative real-time RT-PCR. Values are presented as mean ± SEM of 3–6 observations in each group. *, ** and *** represent *P* < 0.05, *P* < 0.01 and *P* < 0.001, respectively, both for the comparisons indicated by the *horizontal line* and versus NC in the absence of LPS. *ns* non-significant

Treatment with SLPI siRNA reduced both SLPI transcript and protein by about 70% compared to PDL cells treated with non-coding (NC), scrambled construct showing successful knockdown of the SLPI gene (Fig. 3a, b). Basal expression of IL-6 and MCP-1 was increased by 4 and 8 times, respectively, in PDL cells treated with SLPI siRNA (Fig. 4a, b). Stimulation with LPS (1 µg/ml) markedly enhanced both IL-6 and MCP-1 mRNA expression in cells treated with NC as well as in cells treated with siRNA for SLPI. Importantly, however, LPS had a stronger effect on both IL-6 and MCP-1 expression in cells treated with SLPI siRNA than in NC-treated cells (Fig. 4a, b). The relative increase in LPS-induced IL-6 and MCP-1 was not enhanced in SLPI siRNA compared to NC-treated cells (Fig. 4a, b). Treatment with siRNA for SLPI elevated both basal TLR2 and TLR4 expression compared to treatment with the NC construct (Fig. 4c, d). Stimulation with LPS increased TLR4 but not TLR2 transcript in the NC-treated cells (Fig. 4c, d).

### Knockdown of SLPI enhances PDL cell expression of MCP-1 protein

**Fig. 5:**
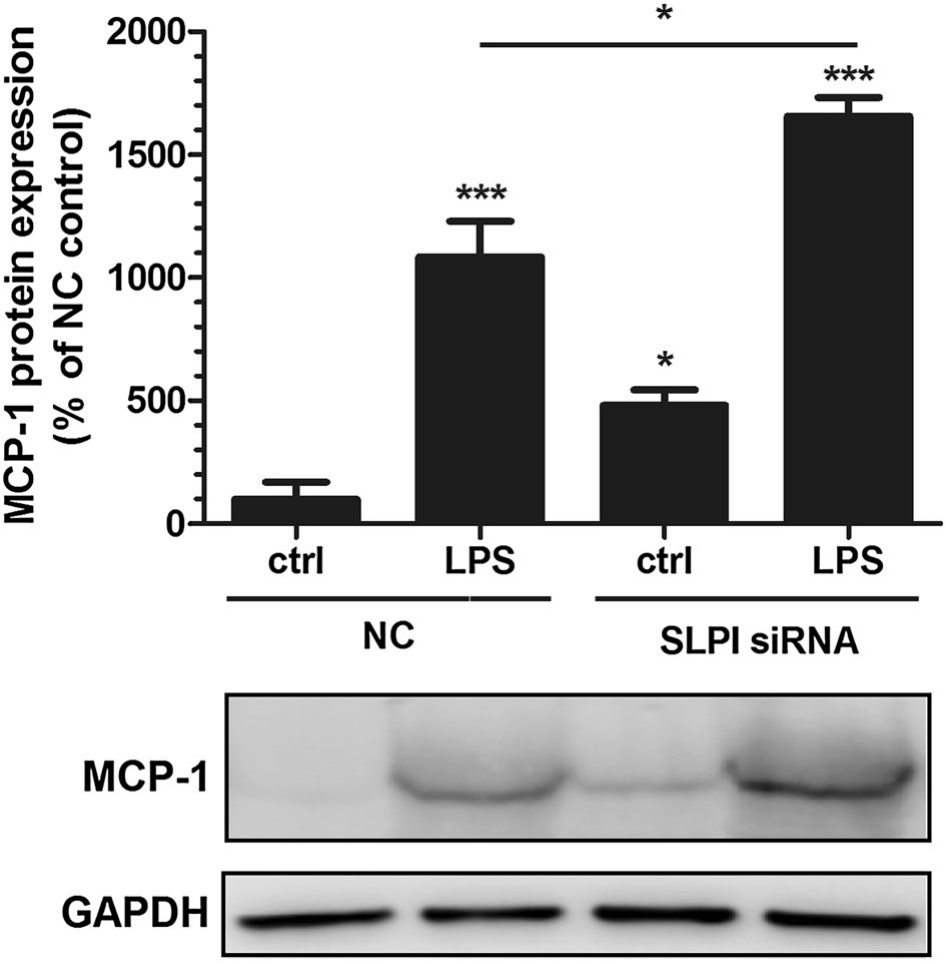
Knockdown of SLPI by siRNA enhances PDL cell expression of MCP-1 protein. Both basal and LPS-stimulated levels of MCP-1 protein are elevated in PDL cells treated with SLPI siRNA compared to NC-treated control cells. The cells were transfected with either SLPI siRNA or NC construct for 96 h and treated with or without 1 µg/ml LPS during the last 24 h of the 96 h transfection period. The MCP-1 protein was determined in PDL cell lysates by Western blotting. MCP-1 is detected as one band at the expected molecular weight (12–13 kDa). The relative MCP-1 concentration was assessed by densitometry and normalized to the GAPDH serving as internal control. Values are presented as mean ± SEM of 4 observations in each group. * and *** represent *P* < 0.05 and *P* < 0.001, respectively, versus NC ctrl. * represents *P* < 0.05 for the comparison indicated by the *horizontal line*

Next, we investigated the MCP-1 protein expression by Western blotting in PDL cells treated with either siRNA for SLPI or NC. Silencing the SLPI gene with siRNA enhanced the basal expression of MCP-1 protein by about 4 times compared with control cells treated with NC (Fig. 5). Stimulation with LPS (1 µg/ml) for 24 h increased the MCP-1 protein by 10 times in NC-treated cells and by 15 times in cells treated with siRNA for SLPI (Fig. 5). Importantly, LPS had a stronger stimulatory effect on MCP-1 protein in the SLPI siRNA-treated cells versus the NC-treated cells, but the relative increase in LPS-induced MCP-1 protein was not more pronounced in siRNA than NC-treated cells (Fig. 5).

### Knockdown of SLPI increases the expression of phosphorylated NF-κB p105 protein in PDL cells

**Fig. 6:**
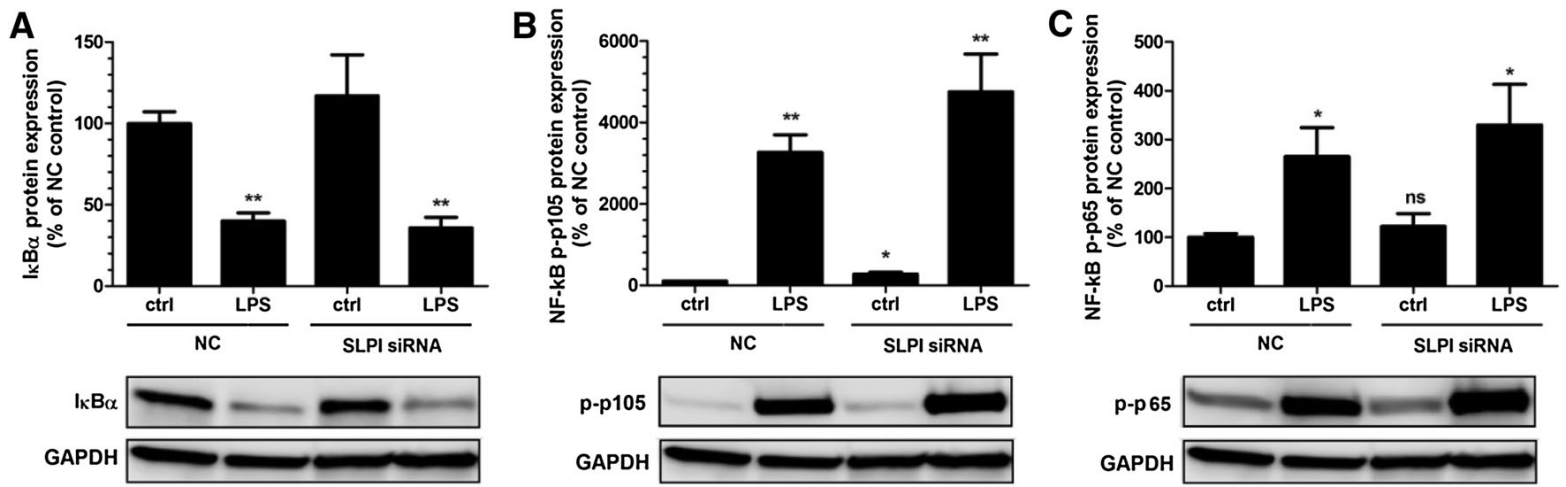
Knockdown of SLPI increases phosphorylated NF-κB p105 protein expression in PDL cells. The cells were transfected with either SLPI siRNA or NC construct for 96 h and treated with or without 1 µg/ml LPS during the last 30 min of the 96 h transfection-period. The IκBα (**a**) and the phosphorylated NF-κB p105 (**b**) and p65 (**c**) proteins were determined in PDL cell lysates by Western blotting at their respective expected molecular weights. IκBα is detected as one band at 37 kDa, phosphorylated NF-κB p105 as one band just over 100 kDa and phosphorylated NF-κB p65 as one band at 65 kDa. The relative IκBα and phosphorylated NF-κB p105 and p65 concentrations were assessed by densitometry and normalized to GAPDH serving as internal control. Values are presented as mean ± SEM of 3 observations in each group. * and ** represent *P* < 0.05 and *P* < 0.01, respectively, versus NC ctrl. *ns* non-significant

SLPI may promote cytokine expression via modulation of NF-κB signaling. Degradation of IκBα and phosphorylation of NF-κB p105 at S933 and NF-κB p65 at S536 are key steps in the activation of NF-κB-regulated gene transcription. Therefore, we assessed expression of IκBα and phosphorylated NF-κB p105 and p65 proteins by Western blotting in PDL cells treated with either siRNA for SLPI or NC and stimulated with or without LPS (1 µg/ml) for 30 min. Knockdown of SLPI had no effect on IκBα expression (Fig. 6a). Stimulation with LPS markedly reduced IκBα protein in both SLPI siRNA and NC-treated cells (Fig. 6a). Basal expression of phosphorylated NF-κB p105 was about three times higher in SLPI siRNA-treated cells compared to NC-treated cells (Fig. 6b). Stimulation with LPS increased the expression of phosphorylated NF-κB p105 severalfold both in cells treated with SLPI siRNA and in cells treated with NC (Fig. 6b). Treatment with SLPI siRNA had no effect on phosphorylated NF-κB p65 expression (Fig. 6c). Stimulation with LPS increased phosphorylated NF-κB p65 protein expression by about three times both in cells treated with SLPI siRNA and cells treated with NC (Fig. 6c).

## Discussion

In the present study, we demonstrate by siRNA, silencing the SLPI gene, that SLPI negatively regulates human PDL cell expression of the pro-inflammatory cytokines IL-6 and MCP-1. Menckeberg et al. [25] reported recently that SLPI determines the microbial responsiveness of human cells, but these data are observed in buccal epithelial cells originating from a different germ layer, i.e., the ectoderm, compared to our data in PDL cells which are cells originating from the mesoderm. We show that SLPI controls PDL cell cytokine expression both on transcript and protein level. Knockdown of SLPI not only enhanced the LPS-induced but also the basal expression of cytokines, suggesting that SLPI suppresses both stimulated and constitutive PDL cell production of cytokines. Extracellular SLPI has been reported to interact with LPS [24], but because we observe that SLPI regulates the basal expression of cytokines we may suggest that SLPI, at least partly, acts intracellular in PDL cells. Treatment with siRNA for SLPI up-regulated not only the cytokines IL-6 and MCP-1 but also the cell surface receptors TLR2 and TLR4 expression, suggesting that SLPI regulates microbial/endotoxin signaling at multiple levels. Importantly, TLR2 is more abundant than TLR4 in the human PDL cells, highlighting the importance of SLPI-induced changes in TLR2 expression [30]. Interestingly, LPS from the periodontitis pathogen *Porphyromonas gingivalis* mainly exerts its effect on host cells via TLR2 [31]. The stimulation of cytokine expression by LPS from *Escherichia coli* (0111:B4), the same LPS used in the present study, has been shown to be similar as that in response to stimulation with LPS from *Porphyromonas gingivalis* in human PDL cells, suggesting that our data are indeed relevant for the in vivo situation [32].

The human PDL cells expressed the SLPI protein but their expression level was lower than for human epithelial HaCaT cells. The HaCaT cells are well known for expressing SLPI and thus included as a positive control [20]. In HaCaT cells, expressing high levels of SLPI, LPS had no effect on IL-6 and MCP-1 expression. On the other hand, in PDL cells, which showed low expression of SLPI compared to HaCaT cells, LPS-stimulation increased both IL-6 and MCP-1 transcript levels severalfold. Thus, we observe a negative correlation between LPS-induced cytokine expression and cellular levels of SLPI. The cell type-specific LPS response in PDL and HaCaT cells may, besides SLPI expression, also reflect that the PDL and HaCaT cells originate from different germ layers and show different cell properties. However, by knocking down SLPI in PDL cells and thereby observe enhanced basal as well as LPS-induced cytokine expression in SLPI knockdown cells, we may conclude that SLPI regulates cytokine expression in PDL cells. Taken together, our data suggest that there is room for both up- and down-regulation of SLPI, thus allowing for SLPI to reduce as well as enhance PDL cell production of pro-inflammatory cytokines.

We show that knockdown of SLPI is associated with modulation of the NF-κB signaling pathway in human PDL cells, suggesting that SLPI regulates PDL cell production of pro-inflammatory cytokines through this mechanism. Silencing the SLPI gene elevated the phosphorylated NF-κB p105 expression, proposing that down-regulation of SLPI may enhance NF-κB activity via this mechanism. SLPI has been shown to directly bind to NF-κB binding sites in the promoter region of the TNF-α and IL-8 genes, thereby blocking NF-κB-induced cytokine expression [21]. Thus, we cannot rule out that down-regulation of PDL cell SLPI also may abolish an SLPI-induced block of NF-κB binding sites in the promoter region of the IL-6 and MCP-1 genes offering another mechanism of action. However, we observe preferentially cytoplasmic and not nuclear SLPI expression in PDL cells, suggesting that SLPI acts through regulation of NF-κB activity rather than via a direct nuclear interaction with the IL-6 and MCP-1 genes.

In summary, we show that SLPI regulates the human PDL cell expression of the pro-inflammatory cytokines IL-6 and MCP-1 and that this effect is associated with modulation of TLR2 and TLR4 expression and the NF-κB signaling pathway. We propose that SLPI represents a novel mechanism that governs the immune cell-like properties of PDL cells, which may be of importance in the pathogenesis of periodontitis.
